# Associations of Circulating Irisin and Fibroblast Growth Factor-21 Levels with Measures of Energy Homeostasis in Highly Trained Adolescent Rhythmic Gymnasts

**DOI:** 10.3390/jcm11247450

**Published:** 2022-12-15

**Authors:** Jaak Jürimäe, Liina Remmel, Anna-Liisa Tamm, Priit Purge, Katre Maasalu, Vallo Tillmann

**Affiliations:** 1 Institute of Sport Sciences and Physiotherapy, Faculty of Medicine, University of Tartu, 51008 Tartu, Estonia; 2 Tartu Health Care College, 50411 Tartu, Estonia; 3 Institute of Clinical Medicine, Faculty of Medicine, University of Tartu, 50406 Tartu, Estonia

**Keywords:** rhythmic gymnasts, irisin, fibroblast growth factor-21, energy homeostasis, training stress

## Abstract

The aim of this investigation was to determine the associations of serum irisin and fibroblast growth factor-21 (FGF-21) with the measures of energy homeostasis, training stress and other energy homeostasis hormones in highly trained adolescent rhythmic gymnasts (RG). Thirty-three RG and 20 untrained controls (UC) aged 14–18 years participated in this study. Body composition, resting energy expenditure (REE), peak oxygen consumption, and different energy homeostasis hormones in serum, including irisin, FGF-21, leptin, and resistin, were measured. Irisin and FGF-21 were not significantly different (*p* > 0.05) between RG and UC groups. In RG, serum irisin was positively associated with REE (*r* = 0.40; *p* = 0.021) and leptin (*r* = 0.60; *p* = 0.013), while serum FGF-21 was related to body fat mass (*r* = 0.46; *p* = 0.007) and leptin (*r* = 0.45; *p* = 0.009). Irisin was related to FGF-21, independent of age, body fat, and lean masses (*r* = 0.36; *p* = 0.049) in RG. In conclusion, serum irisin concentration was associated with energy expenditure and serum FGF-21 level with energy availability measures in lean adolescent athletes, while no relationships of irisin and FGF-21 with energy status measures were observed in lean nonathletic adolescents.

## 1. Introduction

The regulation of energy homeostasis and high training stress is dependent on several peripheral factors that communicate the status of body energy stores to the brain [1]. These peripheral factors are also synthesized from adipose, muscle, and bone tissues, which may act as endocrine organs [2]. For example, it has been found that specific adipose-derived factors, including circulating leptin and adiponectin concentrations, can be sensitive to changes in training volume and could be used to characterize physical stress conditions in athletes [1]. In elite female rowers, Kurgan et al. [3] investigated such peripheral markers as tumour necrosis factor-alpha (TNF-α), interleukin-6 (IL-6), insulin-like growth factor-1 (IGF-1), and leptin to assess variations in energy homeostasis and training stress over a training year. It appeared that fluctuations in training load (high vs. low) were accompanied by parallel changes in TNF-α and IL-6, while IGF-1 and leptin remained relatively stable over a training season in this population of young female athletes with suitable energy availability [3]. Similarly, adipokines such as circulating leptin, adiponectin, resistin, and visfatin concentrations have been used to characterize energy homeostasis in highly trained adolescent rhythmic gymnasts (RG), who begin to exercise at an early age and often adopt negative energy balance to retain lean physique [4,5,6]. Adiponectin was positively associated with weekly training volume in elite young RG participating in World Championships [7], while leptin levels in highly trained adolescent RG can be as low as in anorectic individuals and chronic athletic activity in the presence of prolonged high energy expenditure state decreases leptin concentrations in growing and maturing RG athletes [8]. The importance of tissue crosstalk in energy homeostasis has been highlighted by studies examining the role of different muscle-derived factors in regulating several adipose tissue adaptations to energy metabolism [2,9,10].

Recently, various myokines have been found to mediate training-induced energy and metabolic processes [9,11], besides the most investigated and well-known myokine-IL-6 [1,12]. These myokines include myostatin [13], follistatin [14], irisin [15], and fibroblast growth factor-21 (FGF-21) [16], which have emerged as potential mediators of training-induced energy metabolism. While myostatin is a negative regulator of muscle mass [2], follistatin is a myostatin-binding peptide that promotes skeletal muscle development and exerts metabolic benefits by improving glucose metabolism [14]. One of the more recently identified myokine, irisin, is primarily secreted by muscle tissue and released into circulation during exercise, resulting in increased energy expenditure and improved glucose metabolism [17]. Irisin levels have been reported to be positively associated with body fat mass (FM) as a surrogate measure of energy availability [18]. However, no differences in serum irisin concentrations were observed between amenorrheic athletes, eumenorrheic athletes and nonathletes aged 14–21 years [19], and between normal weight and overweight young women with a mean age of 18 years [20]. Furthermore, serum irisin concentrations were not related to measures of physical activity and physical fitness in a group of healthy lean women of a wide age range [21]. In addition to irisin, FGF-21 has also emerged as an energy homeostasis hormone that has been implicated in the modulation of energy metabolism in athletes [16,22]. Accordingly, FGF-21 has been proposed as a myokine with metabolic effects on glucose and lipid metabolism that promotes body FM loss [2,23]. It, therefore, appears that irisin and FGF-21 may signal energy status in specific groups of individuals. However, the response of these myokines to chronic exercise training remains to be elucidated in lean adolescent females.

The exact role of circulating irisin and FGF-21 levels in energy homeostasis in female athletes is still not clear. We have previously demonstrated that acute negative energy balance caused by prolonged aerobic exercise elicited the increment in serum irisin and FGF-21 levels and the increase in irisin was related to weekly training volume, while the increase in FGF-21 was associated with exercise energy expenditure in young female rowers with a mean age of 18 years [16]. The present study was undertaken to examine the effect of prolonged athletic activity on serum irisin and FGF-21 concentrations in highly trained adolescent RG athletes. To our best knowledge, whether these myokine levels are related to the measures of energy homeostasis, such as body FM as an index of energy stores, resting energy expenditure (REE), training volume, or other hormones involved in energy homeostasis have not been studied in lean adolescent athletes. We hypothesized that serum irisin and FGF-21 concentrations are higher in highly trained adolescent RG in comparison with nonathletes, and secondly that these circulating myokine levels would be associated with other measures of energy homeostasis in highly trained female athletes with chronically increased energy expenditure state.

## 2. Materials and Methods

### 2.1. Participants and Research Design

This study included 53 healthy adolescent females with ages ranging from 14 to 18 years. Participants were divided into rhythmic gymnasts (RG; *n* = 33) and untrained controls (UC; *n* = 20). Before entering the study, participants completed medical and training history questionnaires. Athletes were recruited from local training groups and were competing at the international level. Rhythmic gymnasts had trained regularly for the last 10.3 ± 0.9 years with a mean weekly training volume of 17.6 ± 5.3 h/week. The UC group consisted of adolescents, who took part only in compulsory physical education classes and were not involved in any training groups. Information about the age of menarche, changes in the menstrual cycle, past or present diseases, and any kind of medication, vitamin, or mineral supplement, was collected [24]. None of the participants received any medications or had a history of any chronic diseases. No restrictions were placed on dietary intake, and participants consumed their ordinary everyday diet [25]. All UC adolescent females were eumenorrheic, while 22 participants in the RG group were eumenorrheic and 11 were oligomenorrheic or had secondary amenorrhea. Menstruating participants were examined during the follicular phase, where the blood sample was taken between days 7 and 11 from the onset of menstruation [24].

The study design, purpose, and possible risks were explained to the participants and their parents, who gave their written informed consent before entering the study. The study protocol was approved by the Medical Ethics Committee of the University of Tartu, Estonia and was conducted in accordance with the Declaration of Helsinki. Participants underwent an observational cross-sectional examination. Measurements of the current investigation included anthropometry, body composition, energy expenditure, peak oxygen consumption, and blood analyses.

### 2.2. Measurements

#### 2.2.1. Body Composition

Body height (Martin metal anthropometer, GPM Anthropological Instruments, Zurich, Switzerland) and body mass (medical electronic scale, A&D Instruments Ltd., Abingdon, UK) were measured to the nearest 0.1 cm and 0.05 kg, and body mass index (BMI) was also calculated (kg/m^2^). Body composition was measured by dual-energy X-ray absorptiometry (DXA) using the DPX-IQ densitometer (Lunar Corporation, Madison, WI; USA) Participants were scanned in light clothing while lying flat on their backs with arms on their sides. Whole body fat percent (body fat %), FM, and lean body mass (LBM) values were obtained. All DXA measurements and results were evaluated by the same examiner. The coefficient of variations (CVs) for the obtained results was less than 2% [25].

#### 2.2.2. Resting Energy Expenditure and Aerobic Performance

Resting energy expenditure (REE) was measured in the morning after an overnight fast. Participants were instructed to avoid any intense physical activity for the 24 h period before REE measurement. After voiding, subjects laid down for 15 min before the measurement of oxygen consumption (VO_2_) and carbon dioxide (VCO_2_) production over 30 min. The first 5 min and last 5 min of the measurement were discarded to ensure adequate measurement [26]. A portable open circuit spirometry system (MetaMax 3B, Cortex Biophysic GmbH, Leipzig, Germany) was used, data were stored at 10 s intervals, and the mean of the 20 min was used to calculate REE according to Weir`s equation [27]: Basal metabolic rate (BMR) (kcal/min) = 3.9 [VO_2_ (l/min)] + 1.1 [VCO_2_ (l/min)], and REE (kcal/day) = BMR × 1440 min.

Maximal aerobic performance was determined by a stepwise incremental exercise test until volitional exhaustion using an electrically braked bicycle ergometer (Corival V3; Lode, Netherlands) [28]. The initial work rate was 40 W, and the stage increment was 35 W every 3 min until the maximal voluntary exhaustion was reached. The test was designed to elicit maximal power output at approximately 15–18 min for each subject [28]. Pedaling frequency was set to 60–70 rpm. Participants were strongly encouraged to produce the maximal effort. Respiratory gas exchange variables were measured throughout the test using breath-by-breath mode with data being recorded at 10 s intervals. Subjects breathed through a facemask. Oxygen consumption, carbon dioxide output and minute ventilation were continuously measured using a portable open-air spirometry system (MetaMax 3B, Cortex Biophysic GmbH, Leipzig, Germany). The analyzer was calibrated with gases of known concentration before the test according to the manufacturer`s guidelines. All data were calculated by means of computer analysis using standard software (MetaMax-Analysis 3.21, Cortex, Leipzig, Germany). Peak oxygen consumption was measured, and maximal aerobic performance was defined as described previously [28].

#### 2.2.3. Blood Analysis

Venous blood samples were drawn between 8:00 and 9:00 a.m. after an overnight fast from an antecubital vein with the participants sitting in an upright position. Blood serum was separated and frozen at −80 °C for further analyses. Irisin was determined using an enzyme-linked immunosorbent assay (ELISA) kit using a specific Irisin/FDNC5 monoclonal antibody (R&D Systems Inc., Minneapolis, MN, USA) [29]. This assay had intra- and inter-assay CVs of 2.5% and 8.7%, respectively, and the least detection limit was 0.25 ng/mL. Fibroblast growth factor-21 (FGF-21) was assessed by a commercially available ELISA kit (R&D Systems Inc., Minneapolis, MN, USA) with a minimum detectable level of 1.61 pg/mL, and intra-assay CV 3.5% and inter-assay CV 5.2%. Leptin was determined by Evidence^®^ Biochip Technology (Randox Laboratories Ltd., Crumlin, UK) with the intra- and inter-assay CVs of 4.6% and 6.0%. Resistin was also measured by Evidence^®^ Biochip Technology (Randox Laboratories Ltd., Crumlin, UK) with the intra- and inter-assay CVs of 5.2% and 9.1%.

### 2.3. Statistical Analysis

Data analysis was performed using the SPSS software version 21.0 package for Windows (Chicago, IL, USA). Standard statistical methods were used to calculate means and standard deviations (±SD). Evaluation of data normality was performed with the Kolmogorov-Smirnov method. Data that were not normally distributed were logarithmically transformed prior to analyses to approximate a normal distribution. This was necessary for body FM and serum leptin values. Statistical comparisons between the groups were made using an independent t-test. In addition, effect size (ES, eta squared) thresholds of 0.01, 0.06, and 0.14 were used to identify small, moderate, and large differences, respectively, to define the magnitude of the difference [30]. Pearson correlation coefficients were calculated to assess linear relationships. In addition, partial correlation analyses controlling for age, body FM, and LBM were used to control for confounders [19]. The level of significance was set at *p* < 0.05

## 3. Results

The studied RG and UC groups did not differ (ES < 0.05; *p* > 0.05) in chronological age, body height, body mass, BMI, and REE (Table 1). Age at menarche was higher in RG compared with UC groups, body fat %, FM, and REE/kg were lower, and LBM, training volume, VO_2_peak/kg, and Wmax/kg higher in RG in comparison with UC (*p* < 0.05; ES > 0.12). Although mean serum irisin was not significantly (*p* > 0.05) different between the groups, RG had moderately higher (ES = 0.06) irisin values compared with UC (Table 2). The difference in FGF-21 concentrations between groups was only small in magnitude (ES < 0.05; *p* > 0.05). In addition, leptin levels were largely (ES = 0.34; *p* < 0.0001) and resistin concentrations moderately (ES = 0.06; *p* = 0.077) lower in RG in comparison with UC.

**Figure 1:**
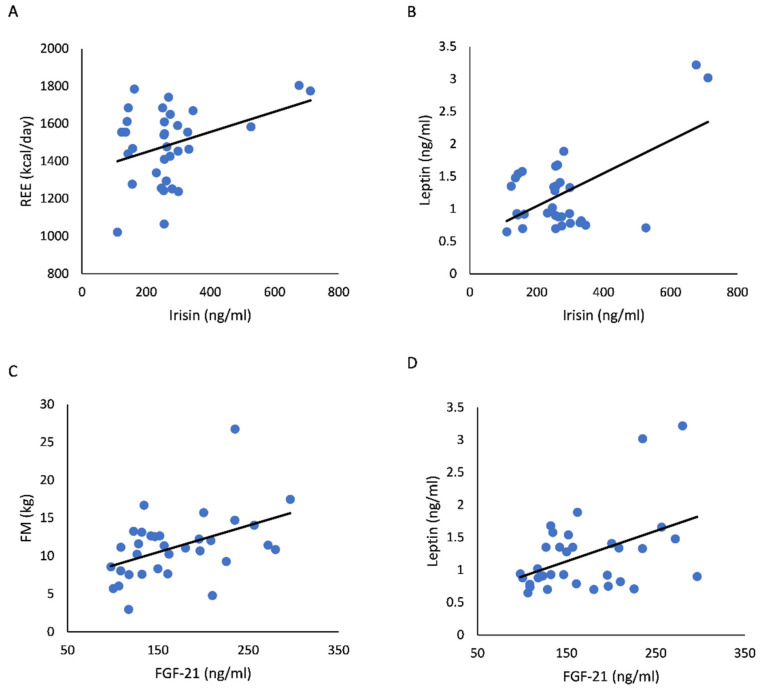
Relationships of irisin levels with resting energy expenditure (REE) (**A**) (*r* = 0.40; *p* = 0.021) and leptin (**B**) (*r* = 0.60; *p* < 0.0001), and relationships of fibroblast growth factor-21 (FGF-21) with body fat mass (FM) (**C**) (*r* = 0.46; *p* = 0.007) and leptin (**D**) (*r* = 0.45; *p* = 0.009) in rhythmic gymnasts.

Table 3 presents correlations of irisin and FGF-21 concentrations with energy measures. In the RG group, serum irisin concentration was positively correlated with REE (*r* = 0.40; *p* = 0.021) and serum leptin level (*r* = 0.60; *p* = 0.013) (Figure 1). In addition, the relationship between irisin and leptin was independent of age, body FM, and LBM (*r* = 0.57; *p* = 0.001). In the UC group, serum irisin concentration was positively correlated to resistin levels (*r* = 0.31; *p* = 0.036), which remained significant after controlling for age, body FM, and LBM (*r* = 0.57; *p* = 0.016). In the RG group, serum FGF-21 concentration was significantly correlated to body FM (*r* = 0.46; *p* = 0.007) and serum leptin levels (*r* = 0.45; *p* = 0.009) (Figure 1), as opposed to the UC group only to leptin (*r* = 0.54; *p* = 0.014). Finally, irisin was related to FGF-21 (*r* = 0.36; *p* = 0.012) only in RG, and the association between irisin and FGF-21 was independent of age, body FM and LBM (*r* = 0.36; *p* = 0.049).

## 4. Discussion

The present study was undertaken to examine the effect of prolonged athletic activity on energy homeostasis regulating hormones, irisin, and FGF-21 in highly trained adolescent RG. We found that serum irisin and FGF-21 concentrations were not significantly different between RG and UC groups. In addition, serum irisin levels were associated with REE and FGF-21 levels with body FM in RG. In contrast, irisin and FGF-21 were not related to energy expenditure and energy availability measures in lean nonathletic UC. These results demonstrate that circulating irisin and FGF-21 levels may play a role in signaling energy status in a setting of a state of long-term high energy expenditure in adolescent athletes.

It has been suggested that the myokine irisin could play an endocrine control of energy metabolism [22,31]. Circulating irisin levels are elevated in obesity as an excess energy state [32] and reduced in anorexia nervosa as a depleted energy state [33], suggesting that irisin levels may reflect energy stores. Indeed, irisin is known to increase energy expenditure by inducing the browning of subcutaneous white adipocytes, which are metabolically favorable for burning energy through thermogenesis [10,17]. Accordingly, irisin is thought to improve glucose and lipid metabolism in response to exercise training [34,35]. Our data linking irisin levels with REE in highly trained adolescent RG are consistent with the literature indicating that irisin may signal energy availability and promote energy expenditure [34]. Similarly, irisin levels were related to REE in young female runners [22]. No such correlation was found in the UC group in our study. One potential explanation for this could be that the UC subjects were in normal body weight and relatively balanced in terms of energy intake and expenditure. Furthermore, irisin concentrations were also not related to different parameters of energy expenditure in patients with anorexia nervosa [10,36]. It could be speculated that the adolescent RG in our study were in a state of subtle energy deficit, as indicated by their reduced body FM and lower measured REE after correcting for LBM, but not in the state of extreme energy deficit observed in anorexia nervosa. In accordance with young female runners [22], no relationship between irisin with body FM was observed in studied RG, while elevated irisin levels have been reported to be independently associated with obesity risk factors, including body FM in obese adolescents [18]. However, significantly lower (ES = 0.34; *p* < 0.0001) leptin concentrations in RG were related to irisin levels, and this association was independent of age, body fat, and lean masses (*r* = 0.57; *p* = 0.001), demonstrating the muscle-adipose tissue crosstalk in energy homeostasis in adolescent lean females with chronic athletic activity. These seemingly conflicting results demonstrate the specificity of irisin interactions with different markers of energy metabolism in various populations and further studies are needed to clarify the exact role of irisin in energy homeostasis. However, the results of our study and that of Singhal et al. [22] would suggest that irisin concentrations may accentuate the increase in energy expenditure in lean adolescent female athletes, as indicated by the positive associations of circulating irisin levels with measured REE in these individuals.

Studies investigating the effects of chronic athletic activity on circulating irisin levels in adolescent athletes are rare. Earlier studies demonstrated that irisin levels were not significantly different between moderately trained young eumenorrheic runners, amenorrheic runners, and nonathletic controls, although irisin levels were the lowest in amenorrheic runners [19]. Another study found that irisin concentrations were lower in young amenorrheic athletes compared with eumenorrheic athletes and nonathletes [22], while a third study in elite male adolescent tennis players did not observe large variations in irisin concentrations over a competitive tournament season, although it was suggested that irisin may modify overall performance during a long-lasting season [37]. In our study, serum irisin levels were moderately, but not significantly higher (ES = 0.06; *p* > 0.05) in RG compared with the UC group. However, although VO_2_peak/kg was higher (ES = 0.12; *p* = 0.012) in RG compared with UC, no relationship between irisin concentration and maximal aerobic performance was observed in RG, similar to previous studies demonstrating that maximal aerobic performance does not influence circulating irisin concentrations in blood [21,38]. In accordance with our results, training volume did not modify circulating irisin concentrations in adult highly trained athletes [15,39] and basal irisin may not be a good marker of training volume over a training macrocycle [38]. However, as interval training caused moderate and significant increases in serum irisin levels in previously untrained adults [40,41], circulating irisin may be a more sensitive marker of training intensity rather than training volume in adult athletes [16,34] as well as in exercising adolescents [42]. It is known that training in rhythmic gymnastics is quite intensive involving numerous jumping exercises daily [6,8] and our studied RG athletes were tested during the preparatory period with a relatively high training volume. It has also been suggested that irisin may be a marker of muscle damage [43] and can provide anti-inflammatory protection [34]. Although the biological role of irisin as a moderator of energy metabolism in response to acute training load remains to be fully elucidated [13], circulating irisin levels have been reported to increase as a result of an acute training session in young female athletes [16]. According to the results of our study, it appears that moderately higher irisin levels in highly trained adolescent RG did not reflect training stress in a setting of chronic high energy expenditure state, whereas decreased irisin levels in amenorrheic athletes likely represent an adaptive response to reduce training stress and conserve energy [22].

In accordance with irisin levels, serum FGF-21 concentrations are higher in obesity [44], reduced in anorexia nervosa [45] and related to body FM [22,23], suggesting that FGF-21 levels may reflect energy stores. Accordingly, 12 weeks of aerobic exercise training decreased circulating FGF-21 concentrations, body mass and glucose uptake in overweight and obese men [46]. It has been suggested that short-term energy expenditure results in increases in circulating FGF-21 levels [47], while long-term chronic energy expenditure may lead to decreased FGF-21 levels to preserve energy [22,48]. Accordingly, acute training load with adequate duration increased serum FGF-21 concentrations in young female athletes [16]. In our study, serum FGF-21 levels were similar between the RG and UC groups in accordance with the previous studies [19,22]. In addition, we found that serum FGF-21 levels were positively correlated with body FM and leptin concentrations in adolescent RG. These relationships suggest crosstalk between muscle and adipose tissue in energy homeostasis and that FGF-21 could be used as a marker of energy stores in adolescent lean females with chronically increased energy expenditure.

Serum FGF-21 concentrations were positively correlated with irisin levels in the RG group. The secretion of FGF-21 and irisin leads to the white adipose tissue browning, uncoupling protein-1-mediated thermogenesis and energy expenditure [19,22]. The secretion of FGF-21 and irisin is increased by the upregulation of peroxisome proliferator-activated receptor-γ, an exercise-induced transcriptional coactivator that promotes energy metabolism [17,49]. Accordingly, our finding of a positive relationship between irisin and FGF-21 suggests a shared pathway for the regulation of energy metabolism in adolescent athletes with high athletic activity.

This study has some limitations. At first, our cross-sectional design rules out the possibility of identifying causal relationships, particularly from the correlation analysis with some individual outliers. Secondly, a relatively small sample size was used, although the number of individuals in both groups was comparable to previous similar studies with athletes in this area [15,19,31,37]. The main strength of the present study is that, to the best of our knowledge, this is the first study investigating whether specific myokine levels such as irisin and FGF-21 are related to the measures of energy homeostasis in highly trained female adolescent athletes as the participants of this study were international level Estonian rhythmic gymnasts from different sports clubs.

## 5. Conclusions

Serum irisin and FGF-21 concentrations were not significantly different between lean adolescent athletes and nonathletic control subjects. Irisin was associated with energy expenditure and FGF-21 with energy availability in lean adolescent athletes with a state of heavily increased energy expenditure, while no relationships of irisin and FGF-21 with energy status measures were observed in lean nonathletic adolescents with normal daily energy expenditure levels.

## Figures and Tables

**Table 1:** Body composition and energy metabolism values (mean ± SD) in rhythmic gymnasts (RG) and untrained controls (UC).

Variable	RG (*n* = 33)	UC (*n* = 20)	*p* Value	ES
Age (yrs)	16.0 ± 1.2	16.5 ± 1.6	0.202	0.03
Age at menarche (yrs)	13.6 ± 1.2	12.5 ± 0.7	<0.0001	0.26
Body height (cm)	166.8 ± 5.3	166.8 ± 5.0	0.976	0.01
Body mass (kg)	55.7 ± 7.0	58.4 ± 7.4	0.180	0.04
BMI (kg/m^2^)	20.0 ± 2.0	21.0 ± 2.2	0.100	0.05
Body fat %	19.5 ± 5.7	30.4 ± 6.2	<0.0001	0.45
Body fat mass (kg)	11.2 ± 4.3	17.8 ± 4.8	<0.0001	0.35
Body lean mass (kg)	42.2 ± 4.1	37.7 ± 3.7	<0.0001	0.25
REE (kcal/day)	1495 ± 208	1520 ± 208	0.669	0.01
REE/kg (kcal/day/kg LBM)	33.4 ± 4.8	38.6 ± 5.0	<0.0001	0.22
Training volume (h/week)	17.6 ± 5.3	2.1 ± 1.3	<0.0001	0.76
VO_2_peak/kg (mL/min/kg LBM)	53.6 ± 7.7	48.4 ± 5.6	0.012	0.12
Wmax/kg (W/kg)	3.2 ± 0.6	2.3 ± 0.4	<0.0001	0.41

ES, effect size (eta squared); BMI, body mass index; REE, resting energy expenditure; VO_2_peak/kg, peak oxygen consumption per kg lean body mass; Wmax/kg, maximal power output per kg body mass.

**Table 2:** Energy homeostasis regulating hormone concentrations (mean ± SD) in rhythmic gymnasts (RG) and untrained controls (UC).

Variable	RG (*n* = 33)	UC (*n* = 20)	*p* Value	ES
Irisin (ng/mL)	272.7 ± 140.0	207.3 ± 113.7	0.084	0.06
FGF-21 (pg/mL)	169.6 ± 56.4	188.1 ± 54.3	0.249	0.03
Leptin (ng/mL)	1.2 ± 0.6	3.7 ± 2.6	<0.0001	0.34
Resistin (ng/mL)	4.6 ± 2.0	5.7 ± 2.4	0.077	0.06

ES, effect size (eta squared); FGF-21, fibroblast growth factor-21.

**Table 3:** Relationships of irisin and fibroplast growth factor-21 (FGF-21) with energy measures in rhythmic gymnasts (RG) and untrained controls (UC).

Variables	Irisin (ng/mL)		FGF-21 (ng/mL)	
	r	*p* Value	r	*p* Value
Fat mass (kg)				
RG	0.25	0.154	**0.46**	**0.007**
UC	−0.07	0.764	0.44	0.054
Lean mass (kg)				
RG	0.29	0.101	0.28	0.144
UC	−0.20	0.398	−0.31	0.187
REE (kcal/day)				
RG	**0.4**	**0.021**	0.26	0.143
UC	−0.05	0.838	0.26	0.27
Training volume (h/week)				
RG	0.14	0.426	−0.34	0.056
UC	0.03	0.886	−0.21	0.365
VO_2_peak/kg (mL/min/kg)				
RG	0.04	0.826	0.19	0.299
UC	0.12	0.618	0.23	0.316
Leptin (ng/mL)				
RG	**0.6**	**<0.0001**	**0.45**	**0.009**
UC	0.07	0.777	**0.54**	**0.014**
Resistin (ng/mL)				
RG	0.31	0.076	0.04	0.808
UC	**0.47**	**0.036**	0.21	0.376

REE, resting energy expenditure; VO_2_peak/kg, peak oxygen consumption per kg lean body mass. Correlations with *p* < 0.05 are listed in bold.

## Data Availability

The data presented in this study are available on a request from the corresponding author for researchers who meet the criteria for access to confidential data.
